# Ventriculoperitoneal Shunt Outcomes of Normal Pressure Hydrocephalus: A Case Series of 116 Patients

**DOI:** 10.7759/cureus.4170

**Published:** 2019-03-04

**Authors:** Eva M Wu, Tarek Y El Ahmadieh, Benjamin Kafka, James Caruso, Salah G Aoun, Aaron R Plitt, Om Neeley, Daiwai M Olson, Robert A Ruchinskas, Munro Cullum, Hunt Batjer, Jonathan A White

**Affiliations:** 1 Neurosurgery, University of Texas Southwestern Medical Center, Dallas, USA; 2 Neurology, University of Texas Southwestern Medical Center, Dallas, USA; 3 Psychiatry, University of Texas Southwestern Medical Center, Dallas, USA

**Keywords:** normal pressure hydrocephalus, nph, ventriculoperitoneal shunt, vps, outcome, predictors, complications

## Abstract

Background

Permanent cerebrospinal fluid (CSF) diversion with a ventriculoperitoneal shunt (VPS) is a treatment option for patients with normal pressure hydrocephalus (NPH).

Objectives

Herein, we examine the outcomes, complication rates, and associations between predictors and outcomes after VPS in patients with NPH.

Methods

This was a retrospective review of 116 patients (68 males, 48 females) with NPH who underwent VPS placement from March 2008 to September 2017 after demonstrating objective and/or subjective improvement after a lumbar drain trial. The Chi-square test of independence was used to examine associations between predictors and clinical improvement after shunting. Complications associated with the VPS procedure were recorded.

Results

The mean age was 77 years (range 52-93). The mean duration of disturbance in gait, cognition, and continence were 29, 32, and 28 months, respectively. Of the 116 patients, 111 followed up at two weeks; of these, improvement in gait, incontinence, and cognition were reported in 72, 20, and 23 patients, respectively. Gait improved more than incontinence or cognition. A shorter duration of gait disturbance predicted an improvement in gait after shunting (p<0.01). Being on a cognition-enhancing medication predicted an improvement in cognition and/or incontinence after shunting (p<0.05). Complications included misplaced proximal catheters (n=6), asymptomatic catheter tract hemorrhages (n=3), bilateral hygromas (n=7), subdural hematomas (SDH) (n=5), and CSF leak (n=1).

Conclusion

VPS placement in patients with NPH is well-tolerated and associated with improved outcomes at least in the short-term follow-up (<6 months). A shorter duration of gait disturbance and being on a cognition-enhancing medication are associated with greater improvement after VPS.

## Introduction

Normal pressure hydrocephalus (NPH) affects 0.5% to 2.9% of elderly patients [1]. Typically, patients present with at least two of the following: gait disturbance, cognitive decline, and urinary incontinence, in addition to ventricular enlargement [2]. The pathological cause of NPH remains unclear and approximately 50% of cases have no identifiable predisposing factors. When a potential diagnosis of NPH is made, a large-volume lumbar tap or a short-term lumbar drain (LD) trial is usually performed to assess the potential response to the cerebrospinal fluid (CSF) diversion. The clinical response to these procedures is often temporary. Therefore, a permanent CSF diversion with a ventriculoperitoneal shunt (VPS) is a treatment option for patients with NPH. An improvement of symptoms is seen in 70% to 85% of patients after shunting [3]; with complication rates up to 35%, LD trials have been shown to have high sensitivity for predicting response to shunting [4]. However, there is no test that can predict with certainty which patients will experience sustained benefit from shunting; therefore, studies to identify factors that can reliably predict shunt outcomes have been conducted [5-7]. The results of such studies, however, remain controversial. The aim of this study is to assess a single center’s experience of outcomes, complications, and associations with predictors after VPS in patients with NPH and to review the current literature.

## Materials and methods

Subjects

NPH patients admitted to the University of Texas Southwestern Medical Center from March 2008 to September 2017 were retrospectively reviewed. A total of 254 NPH patients were admitted for an LD trial. Of these, 116 patients received VPS and are included in this study. Patients who received VPS from other institutions, received shunts for other reasons, or did not undergo a lumbar tap or LD trial were excluded. Institutional Review Board approval was granted for this study.

Preoperative assessment and LD trial protocol

All patients underwent clinical evaluation and neuroimaging in the neurosurgery clinic to assess for a potential NPH diagnosis based on established guidelines [8]. The majority of patients were evaluated by the senior author (JW). All patients who presented with at least two symptoms of the classic NPH triad and ventriculomegaly disproportionate to brain atrophy were considered good candidates for an LD trial. The LD trial was used to support the diagnosis of NPH and to serve as a potential prognostic indicator of the response to permanent CSF diversion. “Responders” of the LD trial were defined as patients who exceeded one-week reliable change indicators or as an improvement by one or more standard deviations on any assessment scale described above or subjective improvement reported by patients themselves and/or family members. The results of the LD trial were discussed with the patients and their families. VPS was offered to responders. Non-responders were referred to neurology for further workup of their functional decline.

Ventriculoperitoneal shunt protocol

After the LD trial, 75 objective responders and 41 subjective responders received a VPS. Postoperatively, patients were admitted overnight for observation and neuroimaging to assess for appropriate proximal catheter placement or evidence of bleeding. Most VPS surgeries were performed using the right frontal approach. Other approaches (right occipital, left frontal, or right parietal) were used based on neurosurgeon preference and considerations, which included prior scalp incisions. A Codman programmable valve (Johnson and Johnson, MA, USA) was used in the majority of cases. Medium pressure Pudenz valves (Integra NeuroSciences, NJ, USA) and Aesculap proGAV valves (Aesculap, Inc., PA, USA) were used in a minority of cases. Hospital length of stay (LOS), complications, or readmissions related to VPS insertion was documented.

Outcome assessment

Potential clinical predictors of VPS outcomes were recorded: age, gender, prior brain or spine surgery, medical co-morbidities, cognition-enhancing medications (anti-Alzheimer (AD) and anti-Parkinson agents), personal or family history of neurodegenerative disease (e.g. Parkinson, AD), as well as the preoperative duration of gait disturbance, cognitive dysfunction, and urinary incontinence. Reasons for prior spine surgery included cervical or lumbar stenosis. The radiographic factors obtained from preoperative brain MRIs included: callosal angle, temporal horn size, and the presence or absence of disproportionate subarachnoid spaces. The callosal angle was defined as the angle between the lateral ventricles as measured on a coronal cut at the level of the posterior commissure [9]. Temporal horn size was defined as the maximal dilation of both temporal horns in millimeters (mm) averaged together [9]. A disproportionate subarachnoid space was defined as the unequal enlargement of the supratentorial CSF spaces. Postoperatively, patients were scheduled to return to the clinic two weeks after surgery to assess wound integrity, remove staples, and evaluate symptoms. The timing of subsequent follow-up visits was determined based upon the degree of improvement, the presence of complications, and the need for valve pressure adjustments. At each clinical encounter, all patients were assessed for clinical and functional improvements in their NPH symptoms. Clinical improvement was assessed with a basic cognitive and gait evaluation (gait was visually assessed for fluidity, speed, and balance). Functional improvement was determined by evaluating the patient’s ability to carry out the activities of daily living and an overall assessment of the patient’s quality of life. The outcomes of NPH symptoms were documented by the attending physician as “better,” “same,” or "worse." Symptoms were classified as “better” if there was sustained improvement in comparison to baseline symptoms reported by patient and/or family members and/or objective improvement in cognitive and gait evaluations performed at follow-up visits. Gait and cognitive outcomes were reported consistently, whereas urinary incontinence was occasionally omitted if it was deemed multifactorial. Neuroimaging was obtained if complications were suspected. VPS settings were adjusted as needed during follow-up visits, depending on symptomatic response or the development of complications (valve pressures were decreased if there was no significant symptomatic improvement or increased in cases of hygromas or low-pressure headaches).

Statistical analyses

Data were entered into an electronic spreadsheet (Microsoft Exel, Microsoft Corporation, Redmond, Washington, US) where dichotomous nominal variables were coded as values 0 or 1 for analysis. The spreadsheet was uploaded into SAS v 9.4 (SAS Institute, Cary NC, USA). A two-sided p-value <0.05 was considered statistically significant. Measures of central tendency were examined to support assumptions of approximately normal distributions for continuous variables. Frequencies and percentages were used to describe all categorical variables. The Chi-square test of independence was used for a comparison of dichotomous variables to examine the associations of clinical improvement after shunting. Dichotomous variables included gender, medical co-morbidities (diabetes, stroke), personal history of neurodegenerative disorders, use of cognition-enhancing medications, prior brain or spine surgery, and disproportionate subarachnoid spaces. Continuous variables, such as age, callosal angle, temporal horn size, duration of gait disturbance, cognitive disturbance, and incontinence, were converted into dichotomous variables based on means.

## Results

Subjects

Between March 2008 and September 2017, 116 patients (68 males, 48 females) with a high suspicion of NPH underwent VPS placement. Patient characteristics are summarized in Table 1. A Codman programmable valve at an initial setting of 100 mmH2O was used in 107 patients. Other initial valve settings were occasionally used based on neurosurgeon preference to avoid the development of hygromas or low-pressure headaches. Shunt types and locations are summarized in Table 2. The mean hospital LOS after VPS placement was 1.8 days. The average duration of follow-up was 1.73 years.

**Table 1:** Characteristics of ventriculoperitoneal shunt patients SD: standard deviation; *: Mean value; VPS: ventriculoperitoneal shunt; MMSE: mini-mental state examination

Patient characteristics	Total number
Total number:	116
Male	68 (59%)
Female	48 (41%)
*Age (years)	77 (SD: 7.4)
*Duration of symptoms (months):	
Gait disturbance	29
Cognitive decline	32
Urinary incontinence	28
Medical co-morbidities:	
Hypertension	77 (66%)
Diabetes	36 (31%)
Stroke	19 (16%)
Congestive heart failure	8 (7%)
Coronary artery disease	30 (26%)
Bleeding disorder	0
Chronic kidney disease	6 (5%)
Neurodegenerative disorders:	
Parkinson	19 (16%)
Alzheimer’s	6 (5%)
Other	5 (4%)
Family history	10 (9%)
Surgical history:	
Spine surgery	29 (25%)
Brain surgery	8 (7%)
Medications:	
Anticoagulant	12 (10%)
Antiplatelet	56 (48%)
Cognition-enhancing	32 (28%)
Antidepressant	52 (45%)
Antiepileptic	10 (9%)
Radiographic findings:	
*Callosal angle (degrees)	70 (32-121)
*Temporal horn size (mm)	6.7 (2.2-17)
Disproportionate spaces	26 (22%)
Objective assessment scores (pre-VPS)	
*MMSE	25.5 (6-30)
*BERG	37 (0-56)

**Table 2:** Characteristics of VPS in patients with NPH VPS: ventriculoperitoneal shunt; NPH: normal pressure hydrocephalus

Shunt characteristics	Total number
Shunt type	
Codman programmable:	107 (92%)
Initial setting of 90 mmH2O	1 (1%)
Initial setting of 100 mmH2O	92 (86%)
Initial setting of 110 mmH2O	5 (4%)
Initial setting of 180 mmH2O	3 (3%)
Initial setting of 200 mmH2O	3 (3%)
Unknown setting	3 (3%)
Aesculap proGAV	6 (5%)
Initial setting 10 cmH2O	6 (5%)
Pudenz – medium pressure	3 (3%)
Proximal shunt location	
Right frontal	106 (91%)
Left frontal	2 (2%)
Right occipital	6 (5%)
Right lateral	1 (1%)
Posterior parietal	1 (1%)

Clinical outcomes after VPS

**Figure 1:**
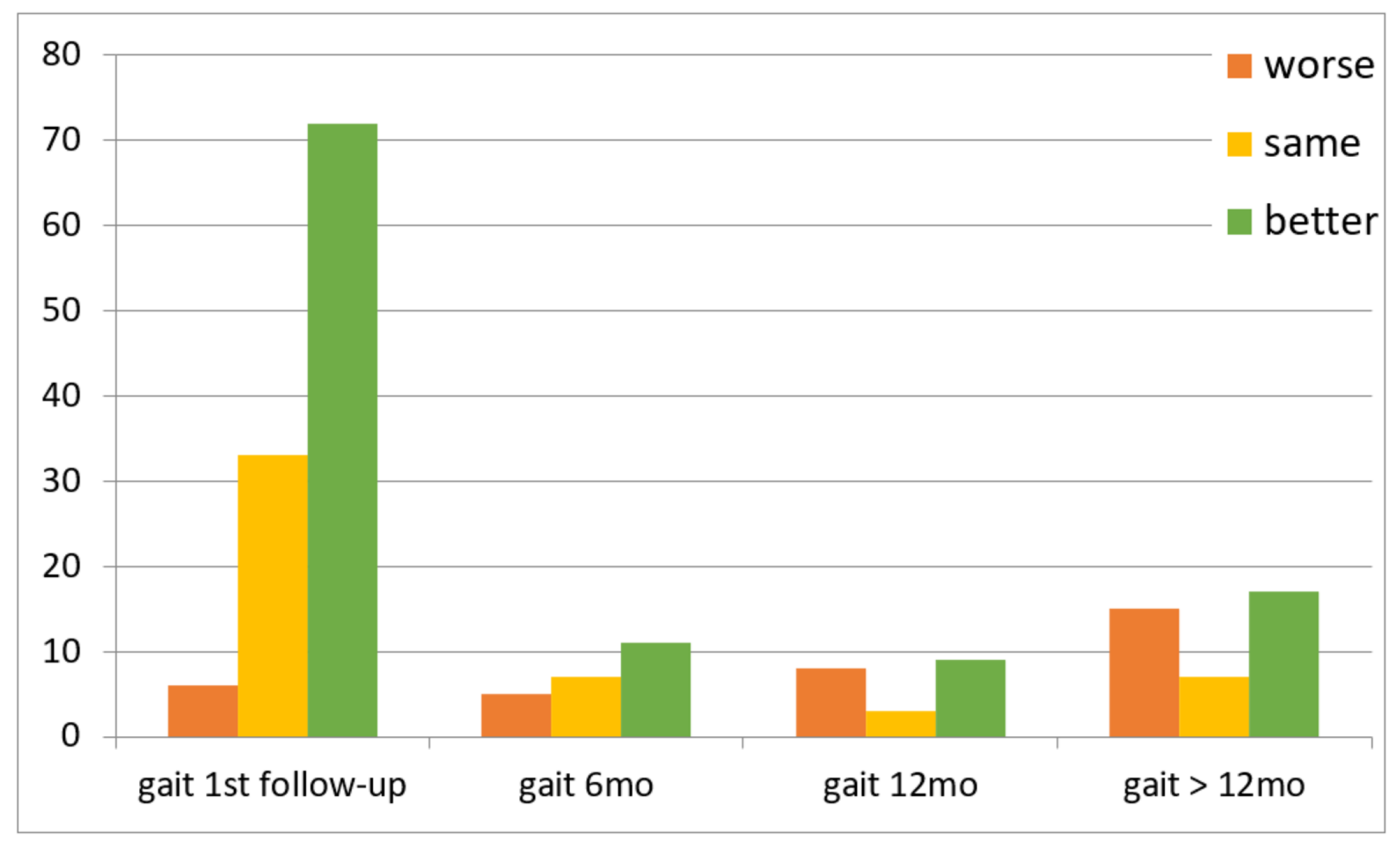
Gait outcomes over time Gait 6mo: outcomes of gait within six months follow-up, gait 12 mo: outcomes of gait between six months and 12 months, gait > 12mo: outcomes of gait after 12 months

**Figure 2:**
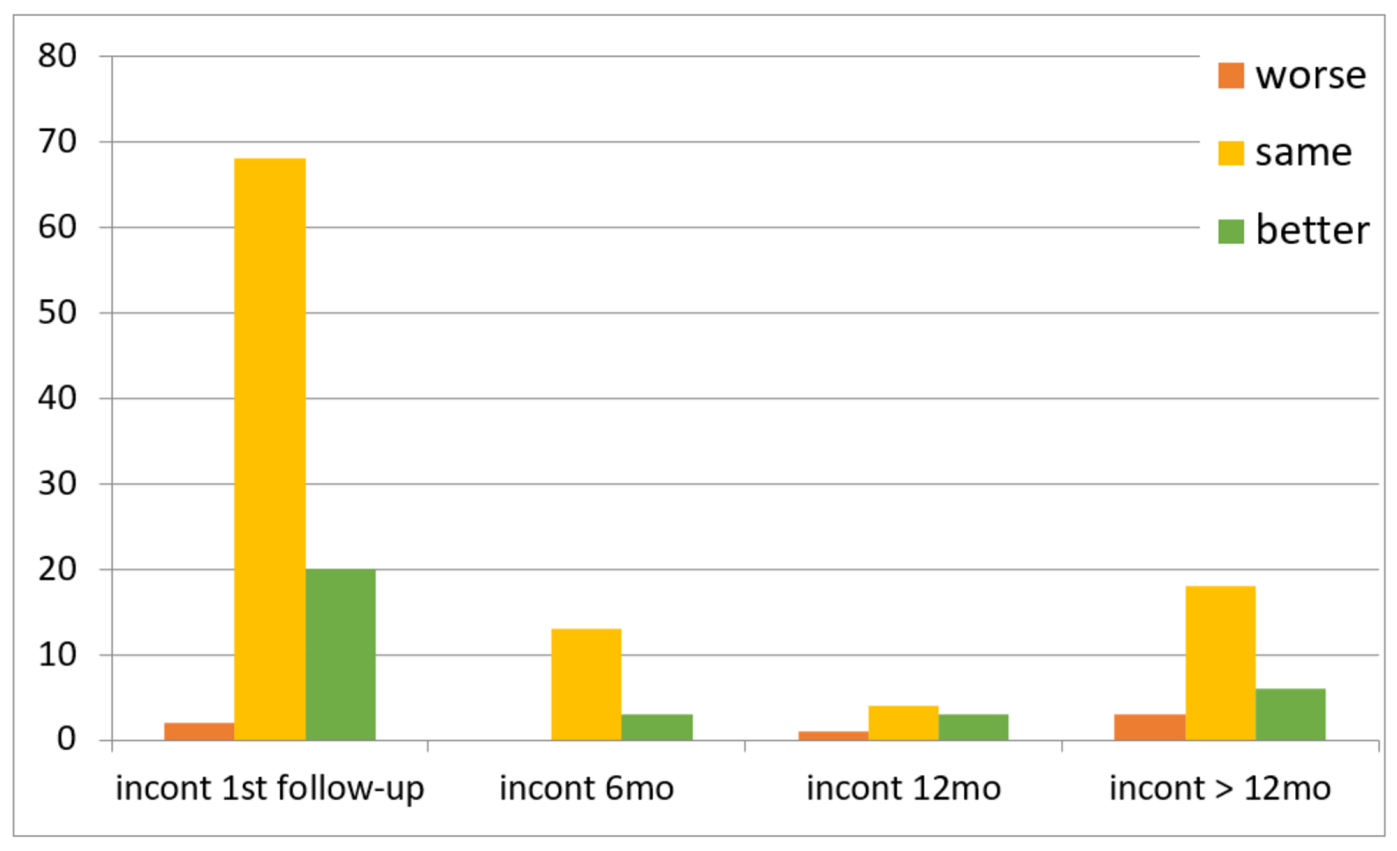
Incontinence outcomes over time Incont 6mo: incontinence outcomes within six months follow-up, incont 12 mo: incontinence outcomes between six months and 12 months, incont > 12mo: incontinence outcomes after 12 months

**Figure 3:**
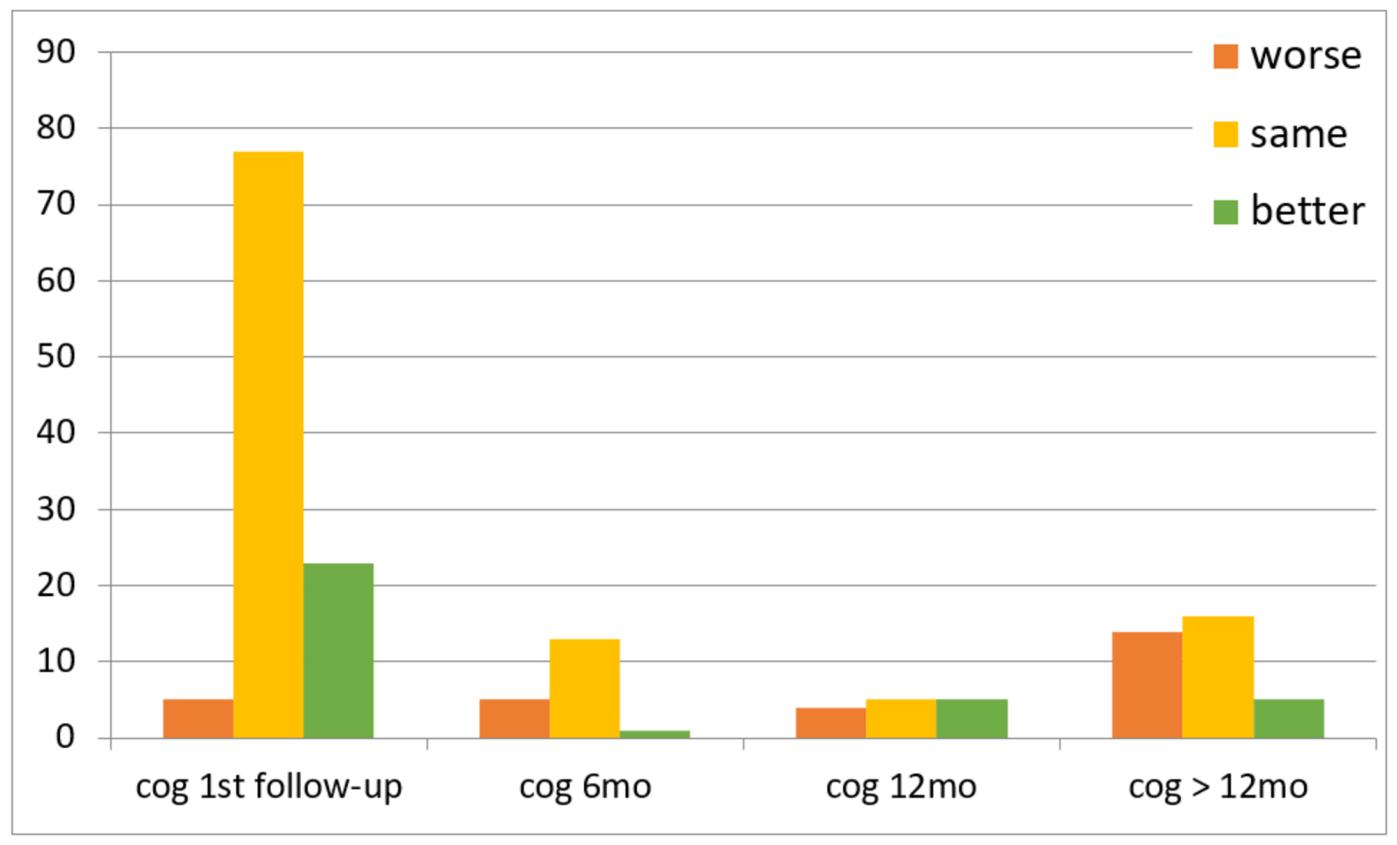
Cognitive outcomes over time cog 6mo: cognitive outcomes within six months follow-up, cog 12 mo: cognitive outcomes between six months and 12 months, cog > 12mo: cognitive outcomes after 12 months

Of the 116 patients, 111 followed up at the two-week timepoint. Five patients missed this initial postoperative assessment but were evaluated within one year of VPS placement. Clinical outcomes over time are summarized in Figures 1-3. In summary, gait was the most likely to improve after shunting (45%, nine of 20 patients) compared to incontinence (37.5%, three of eight patients) and cognition (35.7%, five of 14 patients) at the one-year follow-up.

The Chi-square test of independence was used to investigate associations between predictors of VPS and clinical improvement after VPS. Improvement in gait, incontinence, and cognition was independently compared with each prognostic factor. We found that a gait disturbance of less than 29 months (the mean duration of symptom onset in this patient population) was associated with an improvement in gait after shunting (p=0.0063). The use of cognition-enhancing medications was associated with an improvement in incontinence (p=0.0343) and/or cognition (p=0.0337). None of the other predictors were associated with improvement after shunting. Predictors are summarized in Table 3.

**Table 3:** Predictors of responsiveness after VPS *Duration of gait less than 29 months; **Duration of incontinence less than 28 months; ***Duration of cognitive disturbance less than 32 months; VPS: ventriculoperitoneal shun

	Gait			Incontinence			Cognition		
Predictors of Shunt Responsiveness	Stable / worse	improved	p-value	Stable / worse	improved	p-value	Stable / worse	improved	p-value
Gender:									
Male	16.13% (10)	83.87% (52)	p=0.3368	77.42% (48)	22.58% (14)	p=0.0957	51.61% (32)	61.22% (30)	p=0.4398
Female	25% (12)	75% (36)	p=0.3368	62.50% (30)	37.50% (18)	p=0.0957	60.42% (29)	39.58% (19)	p=0.4398
Age (> 65 years)	21.15% (22)	78.85% (82)	p=0.5976	70.19% (73)	29.81% (31)	p=0.6697	54.81% (57)	45.19% (47)	p=0.6904
Duration of symptoms	0% (0)	100% (23)	p=0.0063*	60% (9)	40% (6)	p=0.3634**	35.29% (6)	64.71% (11)	p=0.1096***
Use of neurostimulants	19.35% (6)	80.65% (25)	p=1.0000	54.84% (17)	45.16% (14)	p=0.0343	38.71% (12)	61.29% (19)	p=0.0337
Diabetes	15.15% (5)	84.85% (28)	p=0.4503	72.73% (24)	27.27% (9)	p=0.8233	42.42% (14)	57.58% (19)	p=0.0944
Stroke	16.67% (3)	83.33% (15)	p=1.0000	66.67% (12)	33.33% (6)	p=0.7774	33.33% (6)	66.67% (12)	p=0.0674
Parkinson	15.79% (3)	84.21% (16)	p=0.7596	57.89% (11)	42.11% (8)	p=0.1773	47.37% (9)	52.65% (10)	p=0.4580
Alzheimer’s	33.33% (2)	66.67% (4)	p=0.3446	50% (3)	50% (3)	p=0.3543	33.33% (2)	66.67% (4)	p=0.4039
Spine surgery	22.22% (6)	77.78% (21)	p=0.7841	70.37% (19)	29.63% (8)	p=1.0000	48.15% (13)	51.85% (14)	p=0.5042
Brain surgery	14.29% (1)	85.71% (6)	p=1.0000	57.14% (4)	42.86% (3)	p=0.4127	28.57% (2)	71.43% (5)	p=0.2384
Callosal angle (>70 degrees)	30.30% (10)	69.70% (23)	p=0.1166	69.70% (23)	30.30% (10)	p=1.0000	60.61% (20)	39.39% (13)	p=0.5340
Temporal horn size (>7 mm)	15.15% (5)	84.85% (28)	p=0.4503	60.61% (20)	39.39% (13)	p=0.1686	48.48% (16)	51.52% (17)	p=0.4041
Disproportionate spaces	12.50% (3)	87.50% (21)	p=0.5378	62.50% (15)	37.50% (9)	p=0.6066	62.50% (15)	37.50% (9)	p=0.4685

Complications

Our overall complication rate of VPS was 19% (22), of which most were asymptomatic and did not require further interventions. Complications are summarized in Table 4. Of the six patients with misplaced proximal catheters, two required revision and repositioning. The three asymptomatic catheter tract hemorrhages were discovered incidentally on routine postoperative brain CT. The five patients who developed bilateral hygromas had an initial shunt setting of 100 mmH2O, which improved with valve pressure adjustments. A unilateral SDH was seen in seven patients with the following initial settings: 100 mmH2O (4), 110 mmH2O (2), and a medium pressure Pudenz (1). One patient required surgical evacuation of the SDH. One patient developed a subcutaneous CSF leak after shunt ligation for low-pressure headaches. The shunt was ultimately removed. There were no mortalities associated with VPS-related complications.

**Table 4:** Complications of VPS placement in patients with NPH CSF: cerebrospinal fluid; VPS: ventriculoperitoneal shunt; NPH: normal pressure hydrocephalus

Complication rates	Total number
Total complications	22 (19%)
Misplaced proximal catheter	6 (5%)
Asymptomatic catheter track hemorrhage	3 (3%)
Bilateral hygromas	5 (4%)
Subdural hematoma	7 (6%)
Distal catheter (CSF leak)	1 (1%)

## Discussion

Clinical outcomes of VPS

A systematic review estimated an overall improvement rate of 71% (range: 33-91%) at three months follow-up and 71% (range: 28-100%) at one-year follow-up [10]. We found that gait (64.9%) was more likely to improve after shunting than cognition (20.7%) or incontinence (18%). Our findings are consistent with previous studies, which concluded that shunting is associated with greater improvement in gait [11-12]. These studies also found that cognitive dysfunction was the least likely symptom to improve after shunting [12]. The low rate of improvement in cognition and incontinence may be due to comorbidities that exist with NPH and difficulties distinguishing between them [13]. Others have reported that gait was the most likely symptom to have sustained improvement over time [3,14]. Pujari et al. [14] concluded that improvement in gait can be sustained for five to seven years. Savolainen et al. [15] reported a sustained improvement in gait in 47% of patients at five years. We found that gait improved significantly above the baseline at the first follow-up visit; however, this response was not maintained at subsequent follow-up visits in a small number of patients, and those who did not see postoperative improvement in gait were less likely to improve on subsequent follow-up visits. Of note, our study was limited in that patients who improved and had sustained improvement may have been less likely to follow up in neurosurgery clinic and, instead, typically continued care with a neurologist. Whether these patients had sustained improvement in the long-term or experienced decline in exam later remains undetermined and is hard to prove given the limitations of a retrospective study. As a result, patients who returned to the clinic for follow-up at six months, 12 months, and after 12 months were those who did not experience symptomatic improvement, had an adverse event, or required valve pressure adjustments. This likely contributed to the low rate of sustained improvement seen in our cohort.

Shunt responsiveness and age/gender

Age has been thought to be a poor prognostic indicator of VPS response; however, its significance as a prognostic factor remains controversial. Our study is consistent with prior studies who reported no significant associations between age and prognosis following VPS [16-17]. The degree of dispersion in our age range gives us more confidence in this finding. However, two other prospective studies concluded that age did influence outcomes after shunting [5,7]. Chang et al. [5] reported that the degree of cognitive improvement after shunting was higher in younger patients. Razay et al. [7] noted that being less than 75 was associated with greater improvement in gait and balance. Age-related neurocognitive decline [18] or the presence of concomitant subclinical neurodegenerative diseases, such as AD or vascular dementia, could explain why age can be a poor prognostic factor. Gender is another predictor that has been debated. Chang et al. [5] found that women experienced more cognitive improvement after shunting. However, our findings are consistent with Caruso et al. [16] and Delwel et al. [6] who found no associations between gender and shunt responsiveness.

Shunt responsiveness and duration of symptom onset

Duration of symptom onset is another predictor that has been investigated. Some studies have found no association between the duration of symptom onset and outcomes after shunting [6,19]. However, other studies have reported the opposite [7,17,20]. The duration of disturbance associated with a positive response to shunting is not agreed upon. Some studies suggest durations of less than six months are most optimal for achieving complete recovery [16], but other studies suggest that symptoms less than 12 months [17] or even 24 months [7] are associated with good outcomes. These studies agree that a shorter duration of symptoms is a positive predictor of outcomes after shunting; however, there were no conclusions made regarding which specific symptom was a predictor. Our findings are partially in line with this literature. We found that a shorter length of gait disturbance was a predictor of positive outcomes after shunting. However, the duration of incontinence and cognitive disturbance were not significant predictors. Some studies concluded that the presence of cognitive disturbance, regardless of duration, predicts poor outcomes after shunting [16]. The discrepancy between our findings and those of others may be explained by different study designs, follow-up protocols, and assessment scoring systems.

Shunt responsiveness and use of cognition-enhancing medications

To our knowledge, there is no literature investigating the use of cognition-enhancing agents as predictors of shunt responsiveness that can be used for comparison with our cohort. A search of the literature revealed only one article describing the outcome of the treatment of NPH associated with Parkinsonism. In this randomized prospective study, treatment with VPS insertion plus oral dopamine therapy was compared to treatment with VPS alone [21]. Patients treated with VPS plus dopamine had greater improvement in the Unified Parkinson’s Disease Rating Scale, Motor section (UDPRS-m) than VPS alone; however, there was no significant difference in MMSE [21]. These findings suggest that the use of dopamine plus VPS may improve motor function but not cognition. Our data show that the use of cognition-enhancing medications (anti-Alzheimer and/or anti-Parkinson agents) predicts improvement in continence and cognition. This finding may be explained by the fact that dementia-associated incontinence is thought to be heavily influenced by the severity of dementia and immobility [22]. It is possible that anti-Alzheimer and anti-Parkinson medications decrease the severity of dementia and immobility, respectively, resulting in an improvement in incontinence. Whether this finding is related to response to medication, shunting, or some synergistic effect is difficult to distinguish and warrants investigation with a randomized control trial.

Shunt responsiveness and medical comorbidities

Medical comorbidities have been thought to be poor indicators of response after shunting. Two studies found that the quantity and type of co-morbidity predicted shunt outcome [23]. Patients were given a comorbidity index (CMI) based on the number and type of co-morbidities. Those with lower CMI scores had better outcomes after shunting. Other studies looking at specific comorbidities concluded that vascular diseases (stroke, ischemic heart disease, and hypertension) were predictors of a worse outcome after shunting [24]. Studies investigating other comorbidities found no association between diabetes and outcome after shunting [24]. Similarly, our findings suggest that a history of diabetes and stroke are not significant predictors.

Shunt responsiveness and neurodegenerative disorders

There are controversial views on the association between AD and VPS outcome. Two retrospective studies reported an improvement in at least one symptom from the baseline in some patients with AD after shunting, suggesting that concomitant AD does not always preclude a positive response after shunting [25]. Other studies have suggested that patients with coexisting AD are less likely to have long-term improvement after VPS [26]. This poor response to shunting may be explained by the irreversible change associated with AD. Our findings are consistent with others who concluded that AD is not a significant predictor of shunt outcome [27].

Shunt responsiveness and radiographic markers

Radiographic markers, such as disproportionate subarachnoid spaces, wide temporal horns, and small callosal angles, have been used in conjunction with clinical findings to aid in the diagnosis of NPH [9]. In our study, none of these markers were significant predictors of shunt response. Our results are consistent with a recent study concluding that callosal angle, temporal horn size, and disproportionate subarachnoid spaces are not predictive of clinical improvement [28], in addition to a study reporting no association between dilated temporal horns and clinical improvement [6]. However, our findings differ from a study reporting that smaller callosal angle, wide temporal horns, and the presence of disproportionate subarachnoid spaces are predictors of good response to shunting [9]. Based on our findings, these radiographic markers may be more valuable as diagnostic tools than predictors of shunt responsiveness.

Complication rates

A recent systematic review of 30 studies (performed after 2006) with a total of 1,573 patients reported an overall complication rate of 8.2% after VPS, of which SDH, infections, intracranial hemorrhages, and mortality accounted for 4.5%, 3.5%, 0.2%, and 0.2%, respectively [10]. A prospective European multicenter study of 142 patients reported an overall complication rate of 28% with hygromas and SDH occurring in 9% and 6%, respectively [29]. Our complication rates for SDH (6%) and hygromas (4%) are comparable to these studies. Furthermore, we included misplaced catheters as a complication, making our overall complication rate higher than that reported in the systematic review [10]. To investigate whether the initial shunt setting was associated with the development of complications of over-drainage, we examined the initial shunt setting in all patients with SDH or hygromas and found that most of these patients had an initial setting of 100 mmH2O. Of note, the majority of patients with an initial setting of 100 mmH2O tolerated it well. A multicenter trial found that using strata shunt valves set at higher drainage pressures (performance level (PL) of 2.5 corresponding to an opening pressure of 140 mmH2O) resulted in similar rates of improvement with less subdural effusions than if starting at lower drainage pressures (PL 1.0, corresponding to an opening pressure of 40 mmH2O) [30]. Taken together, these findings suggest that starting at higher shunt settings is well-tolerated.

Limitations

This is a single-institutional retrospective cohort study limited by missing radiographic and/or clinical data for some patients. Patients lost to follow-up in this cohort may cause a differential attrition bias, resulting in an underestimation or overestimation of our associations. It is difficult to distinguish whether patients with prior spine surgeries had gait difficulty secondary to problems with the spine or normal pressure hydrocephalus. Due to the archival nature of our data derived from a clinical practice setting, another limitation was using different objective assessments on subsequent follow-up visits to compare to the initial preoperative baseline scores. In this elderly population, however, it is burdensome to have patients come back for multiple clinic visits for various clinical assessments by different teams. Given this limitation, we had to classify the outcome of each NPH symptom as “better,” “same,” or “worse” using ratings derived from attending clinic notes. In addition, our study and current literature do not assess the quality of life after shunting. It remains unknown whether shunting impacts the patients’ abilities to carry out daily activities and care for themselves or whether it merely improves their assessment scores and/or perceptions of functioning following VPS. As with other similar investigations, observer and patient ratings are susceptible to bias, which is an inherent limitation of this type of research.

Further investigation with a prospective, randomized controlled study is necessary to determine whether clinical improvement seen after shunting is related to VPS and not a placebo effect. Trials comparing patients treated with a draining VPS to patients with non-draining reservoirs or shunts at very high valve settings is necessary. Conducting such a study, however, remains challenging, given that most patients and their caregivers present to the clinic with a strong desire for VPS after going through extensive neurological workup. Thus, randomization would be very challenging.

## Conclusions

Our study examined the outcomes, complication rates, and associations between predictors of VPS and improvement after VPS in NPH patients. VPS is well-tolerated and is associated with improved outcomes, at least in the short-term follow-up (<6 months). Gait appears to respond better to VPS than to cognition and incontinence. A shorter duration of gait disturbance and being on cognition-enhancing medications are independent predictors of improvement after shunting.

## References

[1] Miyajima M, Kazui H, Mori E, Ishikawa M (2016). One-year outcome in patients with idiopathic normal-pressure hydrocephalus: comparison of lumboperitoneal shunt to ventriculoperitoneal shunt. J Neurosurg.

[2] Yamada S, Kimura T, Jingami N (2017). Disability risk or unimproved symptoms following shunt surgery in patients with idiopathic normal-pressure hydrocephalus: post hoc analysis of SINPHONI-2. J Neurosurg.

[3] Shaw R, Everingham E, Mahant N, Jacobson E, Owler B (2016). Clinical outcomes in the surgical treatment of idiopathic normal pressure hydrocephalus. J Clin Neurosci.

[4] Marmarou A, Young HF, Aygok GA, Sawauchi S, Tsuji O, Yamamoto T, Dunbar J (2005). Diagnosis and management of idiopathic normal-pressure hydrocephalus: a prospective study in 151 patients. J Neurosurg.

[5] Chang S, Agarwal S, Williams MA, Rigamonti D, Hillis AE (2006). Demographic factors influence cognitive recovery after shunt for normal-pressure hydrocephalus. Neurologist.

[6] Delwel EJ, de Jong DA, Avezaat CJJ (2005). The prognostic value of clinical characteristics and parameters of cerebrospinal fluid hydrodynamics in shunting for idiopathic normal pressure hydrocephalus. Acta Neurochirurgica.

[7] Razay G, Vreugdenhil A, Liddell J (2009). A prospective study of ventriculo-peritoneal shunting for idiopathic normal pressure hydrocephalus. J Clin Neurosci.

[8] Halperin JJ, Kurlan R, Schwalb JM, Cusimano MD, Gronseth G, Gloss D (2015). Practice guideline: idiopathic normal pressure hydrocephalus: response to shunting and predictors of response. Neurology.

[9] Virhammar J, Laurell K, Cesarini KG, Larsson EM (2014). Preoperative prognostic value of MRI findings in 108 patients with idiopathic normal pressure hydrocephalus. AJNR Am J Neuroradiol.

[10] Toma AK, Papadopoulos MC, Stapleton S, Kitchen ND, Watkins LD (2013). Systematic review of the outcome of shunt surgery in idiopathic normal-pressure hydrocephalus. Acta Neurochir (Wien).

[11] Bugalho P, Alves L, Ribeiro O (2013). Normal pressure hydrocephalus: a qualitative study on outcome [Article in Portuguese, English]. Arq Neuro-Psiquiatr.

[12] McGirt MJ, Woodworth G, Coon AL, Thomas G, Williams MA, Rigamonti D (2008). Diagnosis, treatment, and analysis of long-term outcomes in idiopathic normal-pressure hydrocephalus. Neurosurgery.

[13] Relkin N, Marmarou A, Klinge P, Bergsneider M, Black PM (2005). Diagnosing idiopathic normal-pressure hydrocephalus. Neurosurgery.

[14] Pujari S, Kharkar S, Metellus P, Shuck J, Williams MA, Rigamonti D (2008). Normal pressure hydrocephalus: long-term outcome after shunt surgery. J Neurol Neurosurg Psychiatry.

[15] Savolainen S, Hurskainen H, Paljärvi L, Alafuzoff I, Vapalahti M (2002). Five-year outcome of normal pressure hydrocephalus with or without a shunt: predictive value of the clinical signs, neuropsychological evaluation and infusion test. Acta Neurochirurgica.

[16] Caruso R, Cervoni L, Vitale AM, Salvati M (1997). Idiopathic normal-pressure hydrocephalus in adults: result of shunting correlated with clinical findings in 18 patients and review of the literature. Neurosurg Rev.

[17] Meier U, Miethke C (2003). Predictors of outcome in patients with normal-pressure hydrocephalus. J Clin Neurosci.

[18] Small SA, Stern Y, Tang M, Mayeux R (1999). Selective decline in memory function among healthy elderly. Neurology.

[19] Hughes CP, Siegel BA, Coxe WS, Gado MH, Grubb RL, Coleman RE, Berg L (1978). Adult idiopathic communicating hydrocephalus with and without shunting. J Neurol Neurosurg Psychiatry.

[20] Petersen RC, Mokri B, Laws ER Jr (1985). Surgical treatment of idiopathic hydrocephalus in elderly patients. Neurology.

[21] Broggi M, Redaelli V, Tringali G (2016). Normal pressure hydrocephalus and Parkinsonism: preliminary data on neurosurgical and neurological treatment. World Neurosurg.

[22] Skelly J, Flint AJ (1995). Urinary incontinence associated with dementia. J Am Geriatr Soc.

[23] Meier U, Lemcke J (2009). Co-morbidity as a predictor of outcome in patients with idiopathic normal-pressure hydrocephalusXIV. Brain Edema XIV.

[24] Boon AJ, Tans JT, Delwel EJ, Egeler-Peerdeman SM, Hanlo PW, Wurzer HA, Hermans J (1999). Dutch normal-pressure hydrocephalus study: the role of cerebrovascular disease. J Neurosurg.

[25] Savolainen S, Paljarvi L, Vapalahti M (1999). Prevalence of Alzheimer's disease in patients investigated for presumed normal pressure hydrocephalus: a clinical and neuropathological study. Acta Neurochir (Wien).

[26] Malm J, Graff-Radford NR, Ishikawa M (2013). Influence of comorbidities in idiopathic normal pressure hydrocephalus - research and clinical care. A report of the ISHCSF task force on comorbidities in INPH. Fluids Barriers CNS.

[27] Bech-Azeddine R, Hogh P, Juhler M, Gjerris F, Waldemar G (2007). Idiopathic normal-pressure hydrocephalus: clinical comorbidity correlated with cerebral biopsy findings and outcome of cerebrospinal fluid shunting. J Neurol Neurosurg Psychiatry.

[28] Kojoukhova M, Koivisto AM, Korhonen R (2015). Feasibility of radiological markers in idiopathic normal pressure hydrocephalus. Acta Neurochir (Wien).

[29] Klinge P, Hellström P, Tans J, Wikkelsø C (2012). One-year outcome in the European multicentre study on iNPH. Acta Neurologica Scandinavica.

[30] Delwel EJ, de Jong DA, Dammers R, Kurt E, van den Brink W, Dirven CMF (2013). A randomised trial of high and low pressure level settings on an adjustable ventriculoperitoneal shunt valve for idiopathic normal pressure hydrocephalus: results of the Dutch evaluation programme strata shunt (DEPSS) trial. J Neurol Neurosurg Psychiatry.

